# Recent Advances in π-Conjugated N^C-Chelate Organoboron Materials

**DOI:** 10.3390/molecules25112645

**Published:** 2020-06-06

**Authors:** Ashanul Haque, Rayya A. Al-Balushi, Paul R. Raithby, Muhammad S. Khan

**Affiliations:** 1 Department of Chemistry, College of Science, University of Hail, Ha’il 81451, Saudi Arabia; 2 Department of Basic Sciences, College of Applied and Health Sciences, A’Sharqiyah University, P.O. Box 42, Ibra 400, Sultanate of Oman; rayya.albalushi@asu.edu.om; 3 Department of Chemistry, University of Bath, Claverton Down BA2 7AY, UK; 4 Department of Chemistry, Sultan Qaboos University, P.O. Box 36, Al-Khod 123, Sultanate of Oman

**Keywords:** boron, π-conjugated materials, opto-electronics, tetracoordinated

## Abstract

Boron-containing π-conjugated materials are archetypical candidates for a variety of molecular scale applications. The incorporation of boron into the π-conjugated frameworks significantly modifies the nature of the parent π-conjugated systems. Several novel boron-bridged π-conjugated materials with intriguing structural, photo-physical and electrochemical properties have been reported over the last few years. In this paper, we review the properties and multi-dimensional applications of the boron-bridged fused-ring π-conjugated systems. We critically highlight the properties of π-conjugated N^C-chelate organoboron materials. This is followed by a discussion on the potential applications of the new materials in opto-electronics (O-E) and other areas. Finally, attempts will be made to predict the future direction/outlook for this class of materials.

## 1. Introduction

The continuing development in the area of π-conjugated materials during the last few decades is the result of the combined interdisciplinary research interests of chemists, physicists, and materials scientists [1]. This class of materials provides an excellent platform to merge the features of organics with inorganics to obtain hybrid materials [2,3]. π-Conjugated materials are of considerable interest to academic and industrial researchers compared to conventional inorganic semiconductor materials, which have involve costs and labor-intensive fabrication procedures. The synthetic versatility, solution processability, and ability to tune their photophysical properties render conjugated organic materials as attractive candidates for applications in various domains of materials science [4]. 

To realize a material with superior optical and electronic properties for real-life applications, one or more strategies, such as ring expansion, π-length extension, and metal or main-group element incorporation into the π-conjugated organic system are being adopted. Among these, the incorporation of a non-carbon dopant is an emerging strategy, as it induces a network of covalent and non-covalent interactions, and allows fine tuning of the frontier orbitals (FOs) and the intramolecular charge transfer (ICT) processes. In the last two decades, a plethora of molecular and polymeric systems incorporating heteroatoms (*viz*. N, Si, S, Se, B, and P) have been reported with intriguing properties and applications. In particular, research on π-conjugated organoboron materials has seen a tremendous upsurge due to high usability in several real-life applications. Both three and four coordinated B-based conjugated materials have been reported. In this review, we wish to present properties and applications of N^C-chelated tetra-coordinated B-based conjugated oligo- and polymeric materials.

## 2. Boron-Bridged π-Conjugated Materials: Properties and Features

Boron (B, atomic number = 5, electronic configuration = 1s^2^ 2s^2^ 2p^1^) is a group 13 element with three valence electrons and two vacant p-orbitals. Depending upon the chelating and auxiliary fragments present, a π-conjugated organic backbone can be attached to the B-center to realize trigonal planar and tetrahedral organoboron compounds. The chelation of organic and inorganic fragments induces rigidity into the framework, along with enhanced π-conjugation and improved photoluminescence (PL) properties [5,6]. Consequently, physio-chemical properties, stability, and applications of the resulting materials can be significantly controlled and tuned by variation of the coordination environments around the B-center. 

The trigonal planar B-fragment is considered to be isoelectronic with the carbocation (sp^2^ hybridization with one unoccupied p_z_ orbital); thus, it is formally electron deficient (Lewis acid). Therefore, when it is incorporated into a π-conjugated framework, there is an overlap between the p orbital of boron and π-orbitals of the organic segment, leading to significant delocalization and PL property modulation [7]. It is interesting that the vacant p-orbital can be both advantageous and detrimental. The benefit of this vacant orbital is that it can be exploited to tune PL properties through Lewis base coordination. On the other hand, low stability against moisture (or other nucleophiles) is also attributed to this vacant orbital. To overcome this challenge, one or more bulky substituents such as 2,4,6-trimethylphenyl (Mes), 2,4,6-tri-*iso*-propylphenyl (Tip), and 2,4,6-*tris*(trifluoromethyl)phenyl (FMes) are often employed. These substituents not only modulate electron density over the material but also sterically shield the boron atom and provide kinetic stability towards air and moisture. Overall, a trigonal planar boron system functions as a strong π-acceptor and a σ-donor [8]. Exploiting these features, a number of main/side chain, cyclic, and Lewis acid-base type B-functionalized materials are available with intriguing properties and applications. 

As mentioned above, coordination of vacant p_z_ orbital of the B-atom paves a unique way to develop new materials with tunable properties. A range of neutral tetracoordinated organoboranes can be obtained by forming covalent bonds with mono-anionic chelate ligands. Tetracoordinated B-units can act as electron-donating substituents. A large number of chelating ligands based on C, N, and O donors have been synthesized and reported by different groups. Unlike three-coordinated compounds, boron atoms here play a more significant role in tetracoordinated boron compounds with the opportunity to fine tune the properties via modulation of electronic and steric effects. The introduction of a boron atom in tetracoordinated disposition within a conjugated framework enhances the planarity of the host (conjugated) structure, leading to improved charge transfer and other properties [9]. Besides, tetracoordinated organoboron structures possess high stability and rigid structure, leading to high fluorescence quantum yields. B ← N coordinate bond is labile compared to the B-F bond; this feature can be exploited to develop molecular switches with on-off PL properties [10]. In the literature, synthesis, characterization, and applications of numerous C^O- [11], N^O [12], N^N [13], N^C [14], C^C [15], and O^O [16] chelated B-containing π-conjugated materials have appeared. These chelating sites come from pyridine, thiazole, iso-quinoline, pyrimidine/quinoline, pyrazine, imidazole, and other cores. 

**Figure 1:**
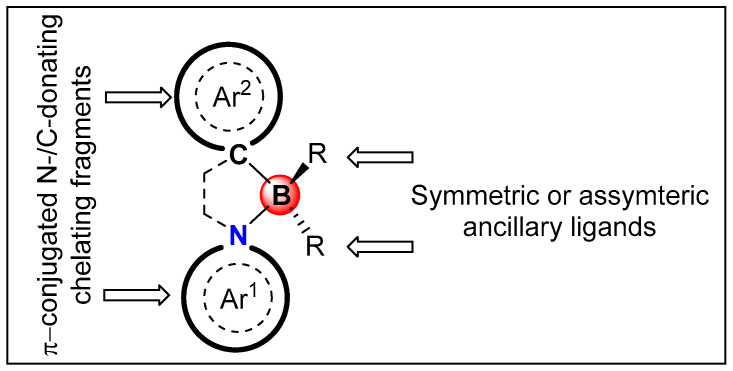
General structure of N^C-chelate organoboron materials discussed in this review. Ar^1^ and Ar^2^ represent aromatic N/C-donating fragments while R represents symmetric or asymmetric ancillary ligands.

In this review, we wish to present the features and properties of N^C-chelate tetracoordinated π-conjugated oligomeric and polymeric materials (Figure 1). In the last two decades, such organoboron-functionalized materials have been extensively studied by the research groups led by Wang, Pischel, and Yam, among others. They discovered several new features and properties associated with these materials. In this review we highlight the recent advances in the new area of N^C chelate compounds because these materials display promising PL features and a specific area has not been reviewed previously. Readers interested in related topics are referred to the reviews [17,18,19,20,21,22,23]. We have divided this review into five main headings. Following this introduction (Section 1), we have briefly compared the features of tricoordinated and tetracoordinated Boron-bridged π-conjugated materials in Section 2. In Section 3, we have discussed the properties of different N^C-chelate-based organoboron compounds. We have reviewed and compared properties of some classical compounds in conjunction with recent ones. Following this, we have outlined some applications of N^C-chelate π-conjugated oligomeric and polymeric organoboron materials in Section 4. In this section, applications such as O-E, imaging, sensing, and other areas have been covered. At the end, in Section 5, we discuss the opportunities and challenges existing in this area of research. 

## 3. π-Conjugated N^C-Chelate Organoboron Materials

**Figure 2:**
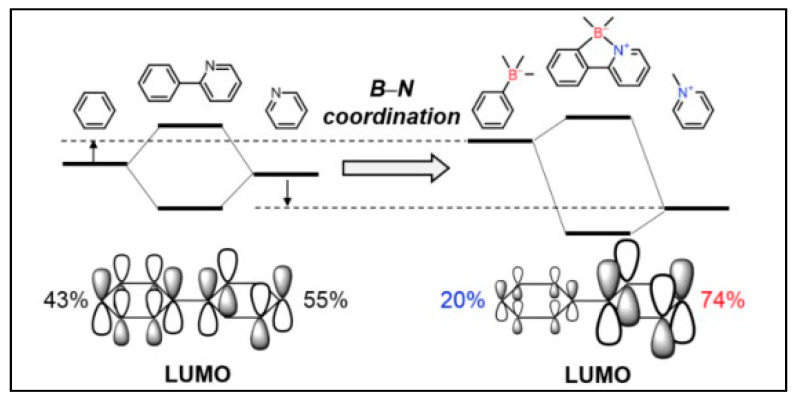
Schematic representation of molecular orbital polarization induced by B-N coordination. Reproduced with permission from reference [24].

Developed around a decade and a half ago, tetracoordinated N^C-chelate organoboron compounds are a unique class of photoresponsive materials. Figure 2 depicts a simple 2-phenylpyridine (ppy) based N^C donor ligand and the effect on the lowest unoccupied molecular orbital (LUMO) energy level upon borylation [24]. As is clear, borylation of a ppy ligand causes polarization of the LUMO. For instance, in non-borylated material, pyridine and phenyl rings contributed ~43% and ~55%, respectively to the LUMO level. Upon B-N coordination, the major contribution was from pyridine ring (~74%) indicating the polarization of the orbitals. This, in turn, led to modulated energy levels and stability (*viz*. destabilization of phenyl π* and the stabilization of pyridine π* orbital) of the compound. In addition, it has also been demonstrated that borylated unit possesses a significantly lower LUMO energy than their non-coordinated counterparts. The replacement of ancillary ligand such as bromine by an aromatic ring such as a thienyl or a phenyl ring has been found to increase the thermal and chemical stability of N^C-chelate organoboron materials [25]. The above-mentioned features motivated researchers to develop new materials with modified C and N-based fully color tunable materials [26,27]. In the subsections below, we discuss properties of different N^C-donor based organoboron compounds.

### 3.1. 2-Arylpyridine-Derived N^C-Chelates

**Figure 3:**
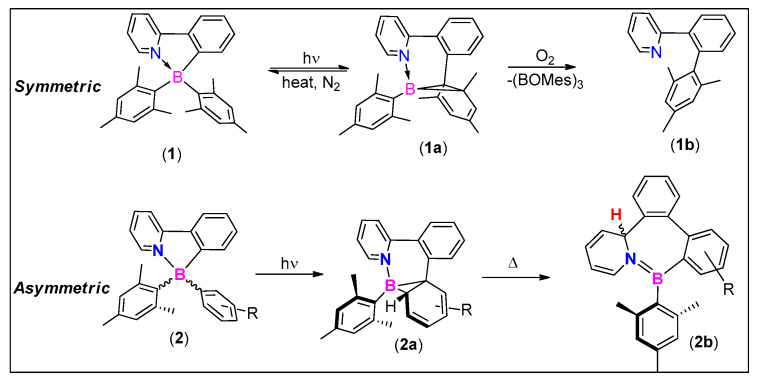
Photoisomerization and oxidation of symmetric and asymmetric organoboron compound ppy-BMes_2_ (Mes = mesityl).

In 2008, Wang and coworkers discovered that the ppy-BMes_2_ organoboron compound (**1**, Figure 3) undergoes intramolecular C-C/C-B bond rearrangement to produce reversible isomerized **1a** and an irreversible isomerized **1b** (Figure 3) products accompanied by a dramatic change in color [28]. Structure property relationship studies using symmetrical and unsymmetrical systems indicated that a bulky substituent (viz. mesityl) on boron fragment is required to initiate the photoisomerization process. In asymmetrical systems (**2**, Figure 3), borirane ring formation takes place regioselectively on the less bulky substituent attached to boron (**2a**; Figure 3). Further thermally assisted transformation produced 4bH-azaborepins (**2b**; Figure 3) [21]. It has been found that in a multi-borylated π-conjugates systems, isomerization takes place at one boron center only while others remain intact and assist this process via ICT [29]. Despite this fascinating process, the rearranged “dark” products significantly limit their usability for real-life applications [30]. 

**Figure 4:**
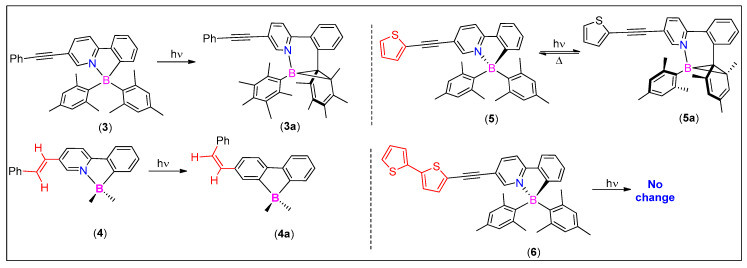
Photoisomerization processes in alkyne and olefin-containing organoboron compounds.

In order to control such process and to increase photochemical stability, several modifications (such as modulation of electronic factors and steric congestion) have been suggested [31,32]. It is shown that the functionalities attached to the chelating/auxiliary units significantly affect the isomerization process [33]. For instance, when a π-conjugated metal acetylide fragment was attached to the pyridine ring, a significant quenching of the photoisomerization (at boron unit) resulted, owing to a low-lying intra-ligand charge transfer (ILCT)/ metal to ligand charge transfer (MLCT) triplet state [34]. On the other hand, conjugation extension using an olefin was found to be an effective strategy as it inhibits photoisomerization (at boron unit) via dissipating energy through the alternative cis-trans isomerization pathway (**3** and **4**, Figure 4) [31]. This cis–trans isomerization of the olefinic bond has also been found to be modulated by metal chelation in such systems [35]. In contrast to this study, authors found that when a bithienyl unit is attached via an alkynyl ligand, it completely turned off photoisomerization arising from the N,C-chelate boryl core (**5** and **6**, Figure 4). They attributed this to relatively low-lying π → π* transition state of the bithienyl unit along with its effective competition with the CT transition of the boryl unit [36]. A similar observation was made by Yam et al. [37] Here, the authors merged photoactive diarylethene-functionalized N∧C chelated thienylpyridine with bis-alkynyl borane complexes.

**Figure 5:**
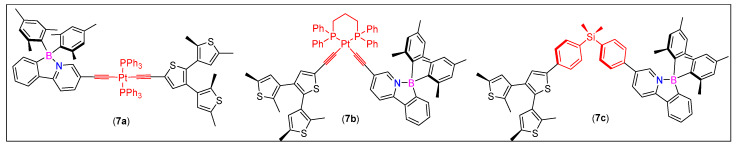
Examples of borylated materials in which two photochromic units are separated by cis, trans-Pt(II)-acetylide, and Si-containing spacers.

In order to examine the competitive photoisomerization processes, Wang and coworkers [38] prepared a series of cis and trans Pt(II) acetylides containing two photochromic units; i.e., dithienylethene (DTE) and B(ppy)Mes_2_ (**7a**–**c**, Figure 5). Interestingly, in such systems, DTE showed preferential reversible photochromism over the boryl unit while the latter enhanced photoisomerization quantum efficiency of the DTE via antenna effect. The quantum efficiency (open → closed) of the system follows the order **7a** < **7b** < **7c**. In order to realize a swift reversible isomerization process, the presence of bulky groups and electron-donating groups has been found to be favorable while the opposite was found for the presence of electron withdrawing groups on the N^C-chelate [39]. Similarly, cyclometallation of the N^C ligands also quenched the photoisomerization process of the B(ppy)Mes_2_ chromophore [40]. Several other studies have been carried out to obtain an in-depth knowledge of such chromophores and more can be found in references [38,41,42,43,44,45,46].

**Figure 6:**
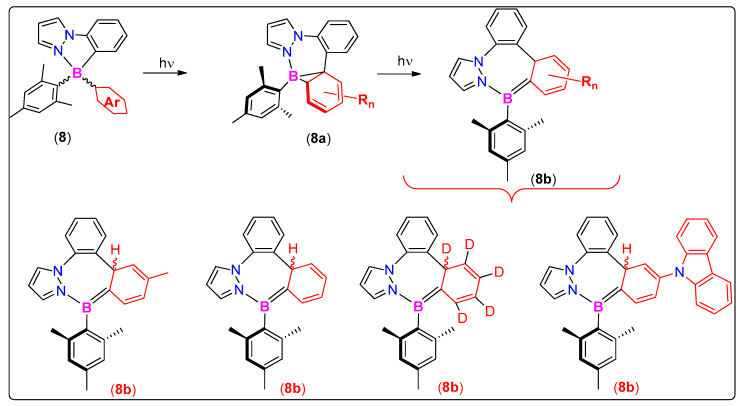
New generation organoboron compounds with rare two-stage photoreactivity.

Through various structural modifications and analogues, it has been established that photoisomerization could also be extended to N^C-chelate in addition to the ppy systems [47]. For instance, in a recent work, Wang and coworkers [47] replaced pyridine by a pyrazole as the *N*-donor (**8**, Figure 6) and reported its two-stage photoreactivity. These new systems undergo photoisomerization to produce thermally stable aza borata bisnorcaradienes (**8a**), which upon prolonged irradiation led to isomerize products 14aH-diazabor-epins or BNN-benzotropilidene (**8b**). Although irreversible, formation of such interesting isomeric products is rare and opens up new vistas for researchers.

### 3.2. 2-Arylthiazole and Aminobenzothiadiazole Derived N^C-Chelates

**Figure 7:**
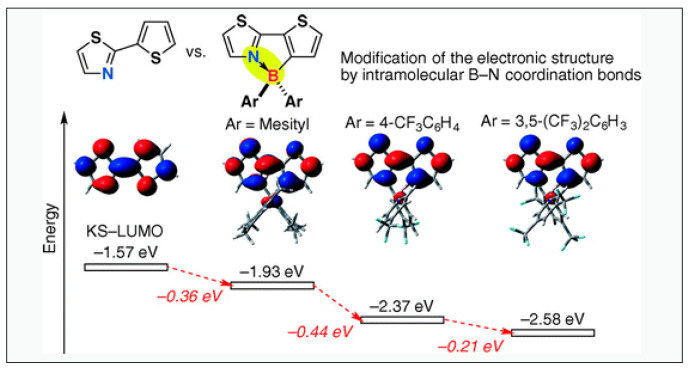
Effect of merging pyridine with phenyl followed by B-N coordination on the LUMO levels. Reproduced with permission from reference [50].

Research work on 2-arylthiazole as the N^C-chelates dates back to 2006 when Yagamuchi and coworkers reported the synthesis and hole mobility properties of mono, di, and oligomeric thienylthiazole-based tetracoordinated B-materials [48]. They found that the incorporation of boryl units into the thienylthiazole core promotes a planar configuration of the π-conjugated framework by intramolecular B-N coordination, which reduces the LUMO level and increases the electron-accepting ability (electron affinity) of the materials. Later they found that the electron affinity along with the thermal stability of the materials can be further improved by replacing thienyl by a C_6_F_4_ moiety and by coordination with (C_6_F_5_)_2_B [49]. For example, Figure 7 shows the effect of borylation on thienylthiazole-based N^C-chelates [50]. As can be seen, the LUMO level of the chelated system reduces upon borylation and further dips upon the modification of ancillary ligands. Similarly, Wang and co-workers reported a decrease in HOMO/LUMO energy level (by 0.6 eV) of a conjugated system upon B-N coordination [51].

**Figure 8:**
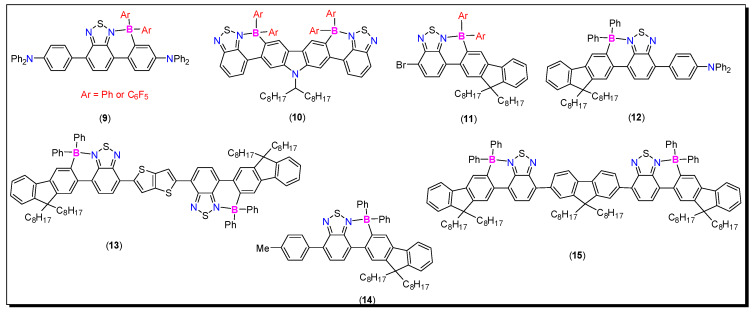
Some examples of BTD-containing N^C-chelate organoboron compounds.

Benzothiadiazole (BTD) is a well-known strong electron-acceptor and is used to develop high-performance donor-acceptor (D-A) materials. BTD-containing materials often possess low band gaps, intense absorption, and emission that extends to the visible to near infrared (NIR) region, and thus are considered as unique materials for opto-electronics (O-E) and other applications [52]. Besides, it has also been reported that HOMO-LUMO energy levels of N^C-chelate organoboron compounds can be tuned via changing the exocyclic boron substituents [53]. Ingleson and coworkers reported simple routes to realize (N^C-chelate)B(aryl)_2_ species (**9**–**15**, Figure 8) [54,55]. They found that the incorporation of borylated aryl amine donor units in compound **9** (Ar = C_6_F_5_) raised the HOMO level, thereby lowering the band gap (~1.5 eV). Despite the fact that the borylated D−A−D material possesses λ_max._ > 700 nm, low PL quantum yields was the main drawback to these systems. 

**Figure 9:**
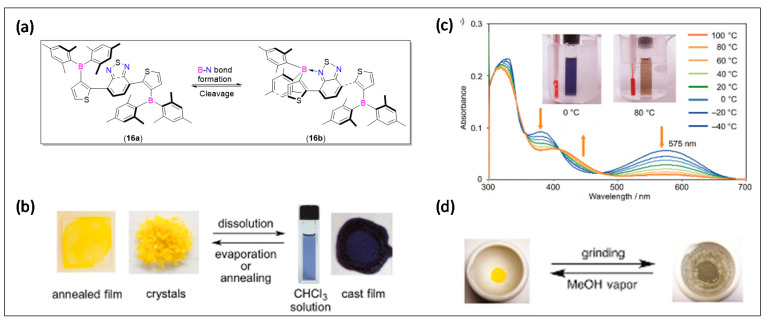
(**a**) Reversible formation of an intramolecular B-N coordination bond in **16a**. (**b**) Photographs of **16a** as an annealed film, yellow crystals, solution in CHCl_3_, and cast film; (**c**) thermochromism of **16a** in toluene; and (**d**) yellow crystals of **16a** before and after grinding. Reproduced with permission from reference [56].

An analysis of the molecular structure of such systems indicated the presence of strain arising from the steric bulkiness of the groups attached to the B-center and the BTD unit. Such a strain can lead to the formation of B-N bonds that can be broken reversibly. Based on this idea, Shimogawa et al. [56] reported a near infrared (NIR) emitting Mes_2_B-substituted BTD material **16** (Figure 9a) having the ability to reversibly form intramolecular B-N coordination bonds. Such reversible bond breaking/formation has a direct effect on the electronic properties and color that are visible to the naked eye. For example, compound **16a**, depending upon its bond formation/cleavage exhibited interesting thermo, solvo, and mechanochromism features (Figure 9b–d).

### 3.3. 2-Arylquinolines-Derived N^C-Chelates

**Figure 10:**
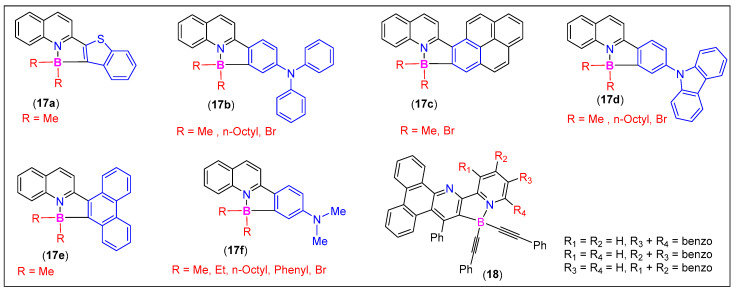
Some examples of 2-arylquinoline-derived N^C-chelates.

Molecular fluorophores capable of absorbing and emitting light at different wavelengths are highly desirable for OE and bioimaging applications. In this quest, Shaikh et al. [57] reported molecular fluorophores based on 2-arylquinoline chelates (**17a**–**f**, Figure 10). The substituents present on the quinolines or on the boron center control the color of the emission. Because of their intense luminescence covering the whole visible region, the fluorophores were used for the imaging of breast cancer cells (MCF 7). Cell viability assays indicated the safe nature (at 1 μM) of organoboron compounds. Extensive bioimaging studies revealed its potential for targeted imaging. Bachollet et al. [58] carried out an extensive structure–optical property relationship study on bipyridine-based tetracoordinated B-materials (BOBIPYs). The new BOBIPYs showed promising properties, such as solid-state luminescence, blue to green emission, and high quantum yields. One of the derivatives based on compound **18** (Figure 10) has the potential to be used as an imaging probe. Other quinoline-based systems with aggregation-induced emission (AIE) properties have also been reported [59].

### 3.4. 1-Arylisoquinolines-Derived N^C-Chelates

**Figure 11:**
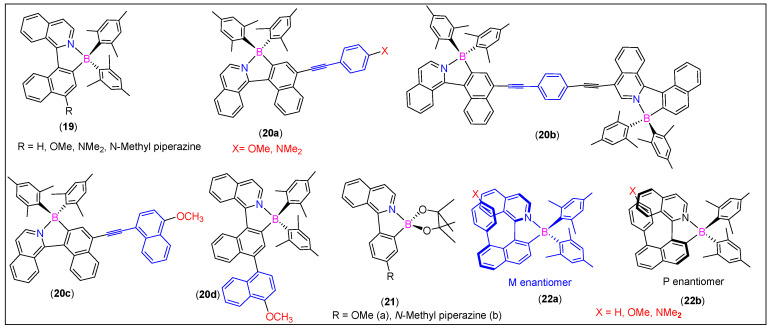
Some examples of 1-arylisoquinoline-derived N^C-chelates.

N^C-chelate organoboron dyes, including those with arylisoquinoline ligands (**19**–**20**, Figure 11), display high stability in air-equilibrated media, and an excited state intramolecular charge-transfer (ICT) character leading to high Stokes shifts, solvatofluorochromic behavior, and high photochemical stability [60,61]. Pischel and coworkers [61] found that the π-extended system **20a**–**c** (Figure 11) exhibits unique two-photon absorption (TPA) properties in the NIR region. In such compounds, the presence of D/A moieties on the termini significantly affected the two-photon absorption cross-section and emission properties. For example, the compound having the donor-π-acceptor (D-π-A) configuration **20a** (X = NMe_2_) showed TPA cross Section 59 GM (960 nm) and 95 GM (700 nm). On the other hand, compound **20b**, having the acceptor-π-acceptor (A-π-A) configuration showed TPA cross Section 14 GM (900 nm) and 223 GM (700 nm). The values higher than their non-extended counterpart **20d** (20 GM at 710 nm and 13 GM at 840 nm) clearly indicated the usefulness of the extension of the π-conjugation via the alkynyl core. 

Similarly to **16a** (Figure 9), arylisoquinoline-based borylated compounds **21a and b** (Figure 11) exhibited a unique steric hindrance-dependent response against temperature and PL properties. These systems exhibited a different geometry in the ICT excited states and thus acted as molecular thermometers [62]. For instance, **21a** has a three-times higher emission quantum yield than the sterically hindered compound. Likewise, the sterically hindered compound showed no temperature effect. In contrast to this, the emission properties of compounds with helicene-type substituents on a borylated arylisoquinoline skeleton **22a and b** (Figure 11) were found to be controlled by the electron-donating substituents on the helicene-type cores [63].

## 4. Applications

Design and development of electronic devices based on conjugated organic materials have seen a huge upsurge in the last few decades. This is due to their synthetic flexibility, excellent film-forming properties, and tunable electronic properties which gained them popularity [64]. As highlighted in the sub-sections above, the PL properties, stability, and applications of B-containing conjugated polymers are greatly determined by the lability of the B-N coordination bond, N^C-donating, π-conjugated cores, and the ancillary ligands. Based on this, several tetradentate B-based small, large, and polymeric π-conjugated materials have been tested for a range of applications. Since we restricted ourselves to N^C based conjugated materials, we highlight some pertinent examples and applications of such materials.

### 4.1. O-E Applications

#### 4.1.1. Organic Light Emitting Diodes (OLEDs)

**Figure 12:**
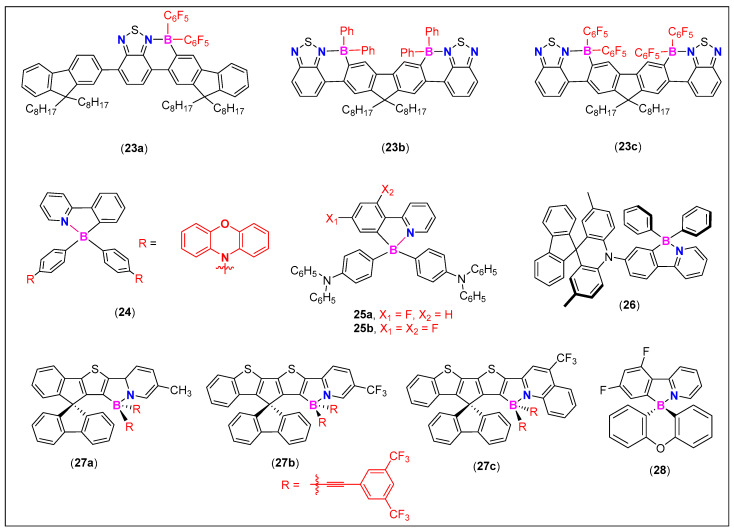
Examples of N^C-donating, π-conjugated cores as OLEDs materials.

For applications as OE components, and sensory or imaging probes, both oligomeric and polymeric systems have been tested. Among these, polymeric materials are favored over oligomeric systems due to manifold benefits. The intense emission extending to the Vis-NIR region, high quantum yield, and charge carrier mobility are some of the important features that make organoborons promising candidates for full-color tunable light emitters [5]. It has been shown that B-N coordination improves EL properties of the luminogens [65]. As discussed before, tetracoordinated B-materials act as electron-donating substituents, and in the presence of a suitable acceptor (such as BT), they create D-A systems with low lying LUMOs. Ingleson and co-workers found that BTD-based D-A materials **23a**–**c** (Figure 12) possess low LUMO energy levels, minimally changed HOMO energy levels, far red/NIR emission with solid state quantum yields of up to 34%, and good stability towards moisture [66]. OLEDs fabricated using these materials showed low turn-on and operation voltages. Among the reported compounds, OLED fabricated using **23a** showed the maximum external quantum efficiency (EQE) value of 0.46% (Table 1) with the EL emission maxima (λ_max_) at 678 nm. The EL value improved slightly (0.48%, λ_max_ = 679 nm) upon changing the device architecture (95:5 wt% PF8BT/**23a**).

**Figure 13:**
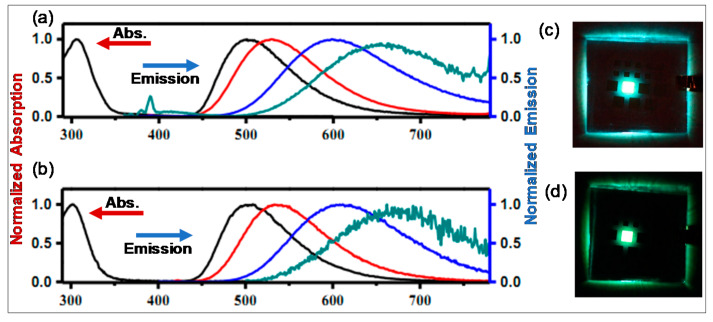
Absorption (arrow towards left) and emission (arrow towards right) spectra of (**a**) **25a**, and (**b**) **25b** in solid (black), and in toluene (red), DCM (blue), and ACN solution (green) at 298 K. Fabricated TADF OLED of (**c**) **25a** (8 wt%) and (**d**) **25b** (25 wt%) devices. Reproduced with permission from reference [67].

Due to their well separated FO, high solid-state quantum efficiency, and small ∆E_S_ → _T_, Wang and coworkers [69] fabricated OLEDs based on compound **24** (Figure 12). However, this material failed dramatically and degraded under operational condition, thereby displaying very poor performance. They suggested that such systems undergo photo-oxidation or exciton-driven transformations within OLEDs, and thus *N*,*C*-chelate organoboron compounds bearing an amino group are not suitable for use as emitters in OLEDs. However, another study proved this finding wrong. By replacing phenoxazine by a *N*,*N*-diphenylamino core (present over ancillary boron) and introducing one or more F-atom on the *C*-donating ppy yielded high quantum yield (56.4–100%) materials **25a**–**b** (Figure 12) with significant delayed fluorescence (Figure 13a,b) [67]. OLEDs that contain dopant **25a** (8 wt%) exhibited an EQE value 20.2%, while those fabricated using **25b** (25 wt%) showed nearly 27% EQE with high current (~64 and 88 Cd/A) and power efficiency (~67 and 82 lm/W) (Figure 13c,d and Table 1). These values (~27%) are considered as some of the best values for thermally activated delayed fluorescence (TADF) materials-based OLEDs. Fluorination not only improved the OLED performance, but also its stability.

Bipolar molecules are considered as a unique class of materials for OE applications as they can transport both the electrons and holes. Despite the fact that bipolar molecules bearing D and A fragments are well known for their ability to enhance TADF, one main challenge in their use as host material is the compression of the band gap via ICT processes, leading to reduction in the device performance. Fortunately, in tetrahedral N^C coordinated materials, the B-atom acts as a node as it separates the FOs, thereby making TADF materials excellent candidates as colorful emissive materials in OLEDs. Matsio and Asuda [24] reported a twisted organoboron TADF molecule **26** (Figure 12), bearing ppy-BPh_2_ as the acceptor and a spiro[2,7-dimethylacridan-9,9′-fluorene] (MFAc) as the D units. OLED fabricated using **26** showed EL an emission maxima (λ_max_) at 494 nm (i.e., intense green color) leading to 22.7% EQE with high current (56.4 Cd/A) and power efficiency (44.3 lm/W, Table 1). On the other hand, devices doped with spiro compounds **27a**–**c** (Figure 12) exhibited tunable EL emission color [68]. Clearly, the presence of different *N*-donating substituents had a marked effect on the emission. For example, a device with dopant **27a** exhibits a blue emission (λ_max_ = 468 nm); **27b** showed a green emission (λ_max_ = 519 nm); and **27c** showed red EL (λ_max_ = 605 nm, Table 1). Recently, Adachi and coworkers found that the OLEDs fabricated using dopant **28** (Figure 12) exhibit a very high efficiency (up to 10.5%), and surpasses the theoretical limit (5%) for conventional fluorescence emitters [70].

#### 4.1.2. Organic Field-Effect Transistors (OFETs)

**Figure 14:**
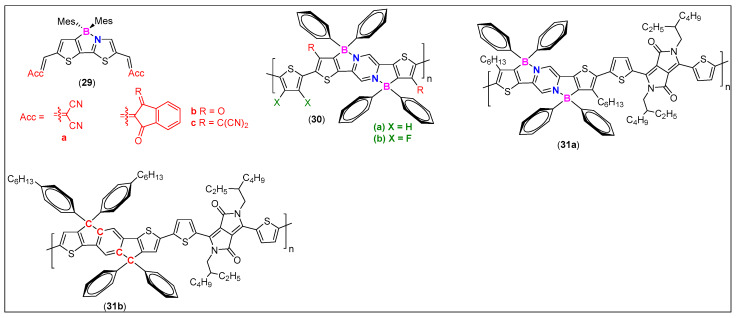
Examples of N^C-donating, π-conjugated cores as OFETs materials.

Organic field-effect transistors (OFETs) have great potential to be developed as next-generation flexible displays, and e-skins. However, compared to p-type semiconductors, a limited number of investigations has been carried out on the n-type materials. Based on the idea that borylation significantly rigidifies the backbone and increases the electron affinity of the resulting materials, new generation OFETs have been developed. For example, Wang and coworkers [71] designed A-π-A type small organic frameworks incorporating indandione and 1,1-dicyanomethylene-3-indandione as acceptors bridged by thienylthiazyl-boron-core (**29a**–**c**, Figure 14). They found that OFETs fabricated using **29a** did not work, which was attributed to the steric hindrance and small size π-core. On the other hand, **29b** and **29c** based semiconducting layers exhibit high performance. In fact, **29c** showed the highest electron mobility value (~1.4 × 10^−2^ cm^2^ V^−1^ s^−1^) reported for small organoboron molecules. They attributed this high performance to increased interchromophoric interactions imparted by the acceptor units with an extended π-surface. Additionally, devices based on **29c** showed much more defined structures and high crystallinity. Besides, control experiments (using C-analogue) indicated that the borylation is an effective way to realize n-type materials [72].

Similar results (structure dependent electron/hole mobilities) were found for semiconductors such as **30** (Figure 14) [73]. It was noted that the transport characteristics (n-type) of fluorinated systems (**30b**) were much better than non-fluorinated ones (**30a**). OFETs studies (top gate/bottom contact TGBC configuration) indicated ambipolar transport characteristics of these materials. Because of the coplanar backbone conformation of the polymers, both **30a** and **30b** show impressive hole and electron mobilities (10^−3^–10^−2^ cm^2^ V^−1^ s^−1^), with **30b** being more effective (~2×) for electron mobility than **30a**. On the other hand, OFET tests indicate a well-defined p-type characteristic for both **31a**–**b** (Figure 14). The hole mobilities of **31b** and **31a** tested by OFETs are 0.059 and 0.035 cm^2^ V^−1^ s^−1^, respectively [74].

#### 4.1.3. Photovoltaics 

Oligo and polymeric π-conjugated N^C-chelate organoboron materials have also been tested as component (donor, acceptor, or dye) in bulk heterojunction (BHJs) and dye sensitized solar cells (DSSCs). As discussed before, science is advancing towards the development of the next-generation organic electronic devices, also called plastic electronics. In the design and development of light to electricity converting devices (solar cells), all-polymer solar cells (all-PSCs) are in great demand. To realize all-PSCs, development of new acceptor materials is one of the most challenging tasks [75]. This is why compared to donor materials, fewer research initiatives have been carried out in the area of acceptor materials.

**Figure 15:**
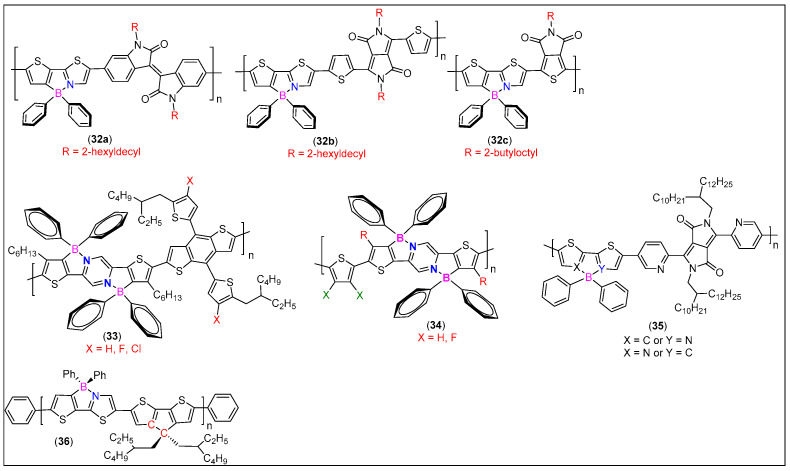
Examples of N^C-donating, π-conjugated cores as BHJ active layers.

Fortunately, N^C-chelate organoboron materials fulfill the requirement of promising acceptor materials due to their unique structures and excellent optoelectronic properties [76]. Several tetracoordinate organoboron materials are available with low band gaps and LUMO levels comparable to PC_61_BM′s LUMO level [72]. While developing materials with high efficiency, Wang and workers [77] found that the electron mobility of a borylated polymer can be significantly modulated by reducing the steric hindrance effect. Using this knowledge, they developed acceptors **32a**–**c** (Figure 15) exhibiting power conversion efficiency (PCE) up to 4.95% (Table 2). Similarly, halogenation is an effective strategy to regulate the performance of PV materials. To understand the effect of halogenation on OE properties, Huang and co-workers [76] reported **33** (Figure 15) with different halogens installed on the donor fragment. This arrangement (BDT as acceptor and halogenated CN borane as acceptor) led to weakened ICT transitions. Depending upon the presence and the type of halogen, downshifted energy levels and varying efficiency were observed. For example, polymer **33** (**X** = **Cl**, η = 4.10%, Table 2) showed higher efficiency than **33 (X** = **F**, η = 3.65%, Table 2), which in turn were better than non-halogenated **33 (X** = **H**, η = 1.54%, Table 2). A further significant improvement in the efficiency was noted when benzodithiophene fragment from **33** was replaced by a thiophene or 3,4-difluorothiophene **34** (Figure 15) [73]. Good coplanarity, narrow bandgap, downshifted energy levels, and extended absorption profiles (350–800 nm) were some of the features found with **34**. A BHJ device fabricated using acceptor **34 (X** = **F**) exhibited a very high PCE (8.42%, Table 2) attributed to the favorable film morphology, balanced charge carriers, and collection among others.

**Figure 16:**
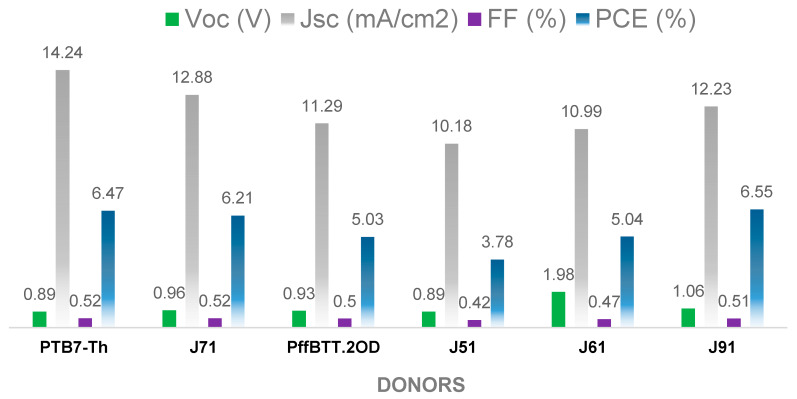
All-PSC device performance of compound **35** with different donors [80,81].

To get in-depth knowledge about different side chains on phase separation morphology and all-PSC device performance, Wang and co-workers [80,81] assessed the performance of regio-random amorphous polymer with large steric hindrance (**35**, Figure 15). The reported acceptor has been found to possess energy level matching that of several donor materials. leading to high performance devices (V_oc_ = 0.89–1.98 V, *J*_sc_ = 10.18–14.24 mA/cm^2^, FF = 3.78%–6.55%, and PCE = 3.8%–6.6%). Among six donor polymers studied, the active layer containing J91:**35** blend showed the most optimal phase separation morphology and the best performance (Figure 16). On the other hand, J51:**35** blend exhibits sub-optimal active layer morphology and poor PV performance (Figure 16). These results indicate that the aggregation tendency in solution of polymer donor is the dominant factor in the phase separation of semi-crystalline polymer donor/amorphous polymer acceptor blend in all-PSCs.

To be used as donor materials, a common strategy is to prepare D-A molecular architecture, which offers tunable LUMO/HOMO energy levels as a low FO level is helpful for harvesting sunlight. Since B-N based materials offer such opportunity, Wang et al. [78] compared the efficiency of polymers **36** (Figure 15). Clearly, the replacement of C–C unit by a B ← N unit significantly altered the frontier energy levels and PV performance. They found that control polymer (all carbon analogue) exhibited higher FOs energy levels, which is detrimental for use in BHJ as donor materials. On the other hand, borylation led to reduction in FOs energy levels, delocalization of FMO throughout the structure, and enhancement in the device performance (PCE up to 3.62%, Table 2).

**Figure 17:**
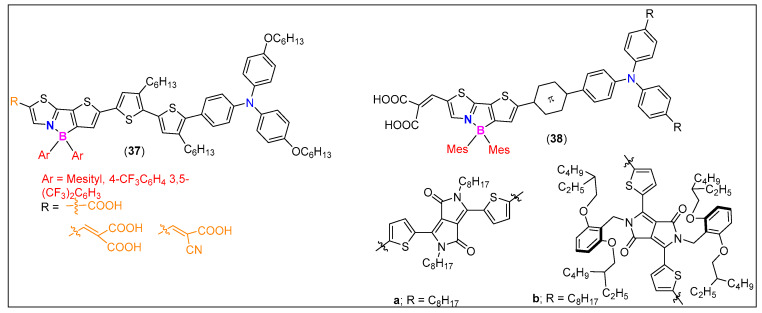
Examples of N^C-donating, π-conjugated cores as DSSC dyes.

Despite the fact that the conversion efficiency of DSSCs has reached >10%, further improvement is necessary. This is especially because of two reasons: one is practical applications on a commercial scale and the second is to develop metal free dyes. In the quest for metal free dyes, Shimogawa and coworkers [50] prepared a series of D-π-A dyes (**37**, Figure 17) as DSSC sensitizers, which contain triphenylamine as electron donors (D), bithiophene as π-spacer, boryl-substituted thienylthiazoles as electron acceptors (A), and carboxylic acid derivatives as anchor groups. DSSCs based on **37** exhibited good PCE values (5.1%–6.1%). Based on these results, they replaced the bithiophene unit by diketopyrrolopyrrole to develop D-π-A dyes **38** (Figure 17). DSSCs containing these dyes exhibited high J_SC_ values (≤19.8 mA cm^−2^) as the absorption of **38** extends into the NIR region. [79]

### 4.2. Sensing

**Figure 18:**
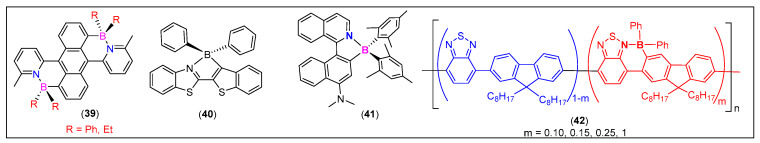
Examples of N^C-donating, π-conjugated cores for sensing and bioimaging.

Due to the highly labile intramolecular N → B-Lewis pair formation dependent PL properties, N^C complexes have also been utilized for the detection of ions in solution and gaseous analytes. In a recent work, Liu et al. [82,83] found that, in the presence of light, red colored BN-functionalized anthracene **39** (Figure 18) rapidly reacts with oxygen to form colorless endoperoxides. Generally, for this type of reaction on typical diarylanthracenes, a photosensitizer is typically required to promote singlet O_2_ generation. However, **39** did not require any catalyst and exhibited self-sensitizing properties. This high, catalyst-free O_2_ reactivity was attributed to strong absorption of visible light, the small singlet-triplet gaps, and the release of steric strain upon peroxide formation. In another report, Schraff et al. [84] showed that in triazole-appended boranes, N-B-coordinate bonds are weak in solution and exist in close (coordinated)-open (non-coordinated) forms. The strength of such bonds (close-open equilibria) can be significantly modulated by changing the substituents present on the triazole ring. The same group showed that this process can be used to monitor on/off emission features upon the addition of different anions [85]. Yan et al. [10] exploited the labile nature of the B ← N coordinate bond and developed a fluorescent probe **40** (Figure 18), which selectively detected fluoride anions (F^−^) in organic solvent. The authors proposed that in the presence of F^−^ anions a competitive reaction (labile B ← N *vs* stable B-F bond) takes place leading to the formation of an open form with quenched emission.

### 4.3. Bioimaging

Bioimaging using a fluorescent chemical probe is an emerging area of research. Several organic, inorganic, and organometallic probes have been reported for the imaging of cellular organelles and biological processes [86,87,88]. Due to their high demand in non-invasive early disease detection and progression, a number of borylated molecular fluorophores have been reported. For example, due to their characteristic features (high chemical and photostability, absorption/emission spanning visible/NIR/FIR regions, biocompatibility, etc.), arylquinolie-based molecular fluorophores emerged as potential candidates [57]. Pischel and coworkers [89] utilized an aryl isoquinoline containing organo-boron N^C chelate dye **41** (Figure 18) for the imaging of the N13 mouse microglial cell line. Moderate absorption co-efficient, large Stokes shift, polarity sensitive fluorescence, and possibility of bioconjugation make them promising imaging probes. Moreover, significant TP absorption cross-sections (up to 61 GM) allow the use of excitation wavelengths in the NIR region (>800 nm). Borylated poly(9,9-dioctylfluorene-alt-benzothiadiazole), **42** (m = 1, Figure 18), is a deep red/near-IR absorbing and highly emissive polymer tested for both OLED and bioimaging applications. [90] Conjugated polymer nanoparticles prepared using pegylated poly(lactic-co-glycolic acid) encapsulating **42** (m = 1) were found to be bright, photostable, low toxicity bioimaging agents for in vivo optical imaging. This includes, but is not limited to, high signal to background ratios, quantum yield (2.3%), low photobleaching (less than 10%), low toxicity, and preferential accumulation in the liver.

### 4.4. Others

In addition to the above-mentioned applications, other applications have been reported too. This includes the activation of small molecules [91,92], switchable chiral anions [93], NLO materials [94,95], among others. In a recent example, Wang et al. [96] reported blue fluorescent polymers exhibiting thermally reversible photochromism. These photochromic polymers, based on photochromic boron chromophores, showed monomer-ratio-dependent photoisomerization quantum efficiencies and boron dependent fluorescence quenching efficiency. The developed material can be used as switchable/erasable ink and are promising candidate for optical device applications.

## 5. Opportunities and Challenges

We highlight the features, properties, and applications of N^C-chelate tetracoordinate π-conjugated borylated materials. As discussed, different types of organic backbone can be utilized as N and C donors in combination with various ancillary ligands to prepare small, medium, and large π-conjugated materials. A thorough review of the literature indicated that a minor change in structure significantly modulates properties; therefore, several new synthetic pathways have been reported. The labile B-N bond formation offers coordination-induced stability to the structure and luminescence enhancement. Complexes such as those based on the phenylpyridine core show interesting reversible “bright” and “dark” photochromic states, while those on pyrazole display rare isomerism (two-stage photoreactivity). Such features can be exploited to develop smart optical devices. Due to their excellent charge transport abilities, especially hole transfer, they are being extensively used for the development of acceptor materials in all-PSCs. Compared to imide and cyano-based polymers, organoboron materials exhibit low performance, which is mainly attributed to the lack of well-designed studies [76]. It is envisioned that the selection of suitable co-units and halogenation at ideal positions are important to further improve the performance [76], which should be considered in future studies.

Some researchers also assessed boron containing complexes as donor materials for BHJs; more research is needed to improve the device performance. Similarly, by modifying π-skeletons, intrinsic D/A fragments, a range of dyes could also be achieved. For the biological applications, it is essential to carry out in vivo studies to explore the pros and cons of the materials in the human body. Besides, to achieve targetability, probes bearing a targeting unit (such as peptides) should also be prepared and assessed.

## 6. Conclusions

This article provides an overview of π-conjugated N^C-chelate organoboron materials. We delineated the important properties, features, and applications of different classes of such materials. We discussed here the effects of different structural components on the properties of the materials. The examples selected in this article clearly demonstrate that tetracoordinate N^C-chelate organoboron materials are potential candidates for the development of next generation materials. We have also suggested some future directions which might prove helpful for the design and development of new boron-based materials for modern applications.

## Figures and Tables

**Table 1:** OLED performances of different π–conjugated N^C-chelate organoboron materials.

Comp. #	Device Architecture	EQE (%)	Current Efficiency (Cd/A)	Power Efficiency (lm/W)	Ref.
**23a**	ITO/Plexcore OC/PF8-TFB/PF8-BT/PF8-TFB/**23a**)/Ba	0.46	-	-	[66]
**23b**	ITO/Plexcore OC/PF8-TFB/PF8-BT/PF8-TFB/**23b**)/Ba	0.14	-	-	[66]
**23c**	ITO/Plexcore OC/PF8-TFB/PF8-BT/PF8-TFB/**23c**)/Ba	0.13	-	-	[66]
**25a**	ITO/PEDOT:PSS/TAPC/mCP/mCPCN doped with **25a**)/3TPYMB/LiF/Al	20.2	63.9 ^a^	66.9 ^a^	[67]
**25b**	ITO/PEDOT:PSS/TAPC/mCP/mCPCN doped with **25b**)/3TPYMB/LiF/Al	26.6	88.2 ^b^	81.5 ^b^	[67]
**26**	ITO/HAT-CN/α-NPD/CCP/EML/PPF/TPBi/Liq/Al	22.7	56.4	44.3	[24]
**27a**	ITO/PEDOT:PSS/**27a**/MCP/3TPYMB/TmPyPB/LiF/Al	1.1	1.6	1.0	[68]
**27b**	ITO/PEDOT:PSS/**27b**/MCP/3TPYMB/TmPyPB/LiF/Al	1.3	4.8	3.0	[68]
**27c**	ITO/PEDOT:PSS/**27b**/MCP/3TPYMB/TmPyPB/LiF/Al	0.9	1.4	0.9	[68]

^a^ = Peak value at 8 wt% concentration; ^b^ = Peak value at 25 wt% concentration; ITO = indium tin oxide; OC = organic conductive; PF8-TFB = poly[(9,9-dioctyl-fluorenyl-2,7-diyl)-co-(4,40-(*N*-(4-sec-butylphenyl)-diphenylamine)]; PF8-BT = poly[(9,9-di-n-octyl-fluorenyl-2,7-diyl)-alt-(benzo[2,1,3]-thiadiazol-4,8-diyl)]; PEDOT:PSS = poly (3,4-ethylenedioxythiophene): poly(styrenesulfonate); TAPC = di-[4-(*N*,*N*-ditolylamino) phenyl] cyclohexane; mCP = *N*,*N*-dicarbazolyl-3,5-benzene; mCPCN = 9-(3-(9*H*-carbazol-9-yl)phenyl)-9*H*-carbazole-3-carbonitrile; 3TPYMB = tris-[3-(3-pyridyl)mesityl]borane; LiF = lithium fluoride; HAT-CN = 1,4,5,8,9,11-hexaaza triphenylenehexacarbonitrile; α-NPD = 4,4′-bis-[*N*-(1-naphthyl)-*N*-phenylamino]-1,1′-biphenyl; CCP = 9-phenyl-3,9′-bicarbazole; EML = emission/emitting layer; PPF = 2,8-iso (diphenylphosphoryl) dibenzo[b,d]furan; TPBi = 1,3,5-tris(*N*-phenylbenzimidazol-2-yl)-benzene; Liq = 8-hydroxyquinoline lithium; MCP = *N*,*N*’-dicarbazolyl-3,5-benzene; TmPyPB = 1,3,5-tri[(3-pyridyl)-phen-3-yl]benzene.

**Table 2:** PV (all-PSCs and DSSCs) performances of selected π-conjugated N^C-chelate organoboron materials.

Comp. #	Device Architecture	V_oc_ (V)	*J*_sc_ (mA/cm^−2^)	FF (%)	PCE ^a^ (%)	Ref.
**32a**	ITO/PEDOT:PSS/**PTB7-Th:32a**/Ca/Al	0.92	11.37	48	4.95	[77]
	ITO/PEDOT:PSS/**PTB7:32a**/Ca/Al	0.93	9.05	45	3.71	
**32c**	ITO/PEDOT:PSS/**PTB7-Th:32c**/Ca/Al	1.08	0.51	22	0.10	
	ITO/PEDOT:PSS/**PTB7:32c**/Ca/Al	1.00	2.48	30	0.63	
**33**	ITO/PEDOT:PSS/**PBDB-T:33**/PDINO/Al	0.97 (X = H) 0.95 (X = F) 0.95 (X = Cl)	4.15 8.74 9.19	38.12 43.66 46.55	1.54 3.65 4.10	[76]
**34**	ITO/PEDOT:PSS/**PBDB-T:34**/PNDIT-F3N/Al	0.92 (X = H) 0.92 (X = F)	8.01 13.01	48.7 69.8	3.79 8.42	[73]
**36**	ITO/PEDOT:PSS/**36:PC_71_BM**/LiF/Al	0.82	9.89	46.1	3.62	[78]
**37**	**37**/TiO_2_	0.51–0.73	10.3–14.2	54–72	3.9–6.1	[50]
**38**	**38**TBP/TiO_2_ or **38**/TiO_2_ or **38**+DCA-TBP/TiO_2_ or **38**+DCA/TiO_2_	0.44–0.68	7.8–19.8	49–68	3.2–6.1	[79]

^a^ = average PCE value. PDINO = 2,9-Bis[3-(dimethyloxido amino)propyl]anthra[2,1,9-def:6,5,10- d′e′f′]diisoquinoline-1,3,8,10(2H,9H)-tetrone; PNDIT-F3N = poly[[2,7-bis(2-ethylhexyl)-1,2,3,6,7,8-hexahydro-1,3,6,8-tetraoxobenzo[lmn][3,8]phenanthroline-4,9-diyl]-2,5-thiophenediyl[9,9-bis[3-(dimethylamino)propyl]-9H-fluorene-2,7-diyl]-2,5-thiophenediyl]; PTB7-Th = poly[4,8-bis(5-(2-ethylhexyl)thiophen-2-yl)benzo [1,2-b:4,5-b’]-dithiophene-co-3-fluorothieno[3,4-b]thiophene-2-carboxylate]; PTB7 = poly({4,8-bis[(2-ethylhexyl)oxy]-benzo[1,2-b:4,5-b’]dithiophene-2,6-diyl}{3-fluoro-2-[(2-ethylhexyl)carbonyl] thieno[3,4-b]thiophenediyl}); PBDB-T= poly[(2,6-(4,8-bis(5-(2-ethylhexyl) thiophen-2-yl)-benzo[1,2-b:4,5-b′]dithiophene))-*alt*-(5,5-(1′,3′-di-2-thienyl-5’,7′-bis(2-ethylhexyl)benzo [1′,2′-c:4′,5′-c′]dithiophene-4,8-dione)]; PC_71_BM = [6,6]-phenyl-C_71_-butyric acid methyl ester; TiO_2_ = titanium(IV) oxide or titanium dioxide; DCA = deoxycholic acid; TBP = 4-*tert*-butylpyridine.
